# Supplementary material for: Polymerase-guided base editing enables in vivo mutagenesis and rapid protein engineering
Source: Nat Commun. 2021 Mar 11;12:1579. doi: 10.1038/s41467-021-21876-z (PMC7952560; doi:10.1038/s41467-021-21876-z)
Supplement: Supplementary file 1 — Supplementary Information [file 41467_2021_21876_MOESM1_ESM.pdf]

Supplementary Materials

Title: Polymerase-guided base editing enables *in vivo* mutagenesis and rapid protein engineering

Authors:

Aaron Cravens[1,5], Osman Jamil[2,5], Deze Kong[1], Jonathan T. Sockolosky[3], Christina D. Smolke[1,4]

Affiliations:

[1]Department of Bioengineering, 443 Via Ortega, MC 4245, Stanford University, Stanford, California 94305, USA
[2]Department of Chemical Engineering, 443 Via Ortega, MC 4245, Stanford University, Stanford, California 94305, USA
[3]Departments of Molecular and Cellular Physiology and Structural Biology, Stanford University School of Medicine, Stanford, CA 94305, USA
[4]Chan Zuckerberg Biohub, San Francisco, CA 94158, USA
[5]These authors contributed equally: Aaron Cravens, Osman Jamil

**Correspondence should be addressed to Christina D. Smolke*
Phone: 650.721.6371
FAX: 650.721.6602
E-mail: csmolke@stanford.edu

This PDF file includes:

Supplementary Figures 1-19
Supplementary Tables 1-5

Supplementary Figures

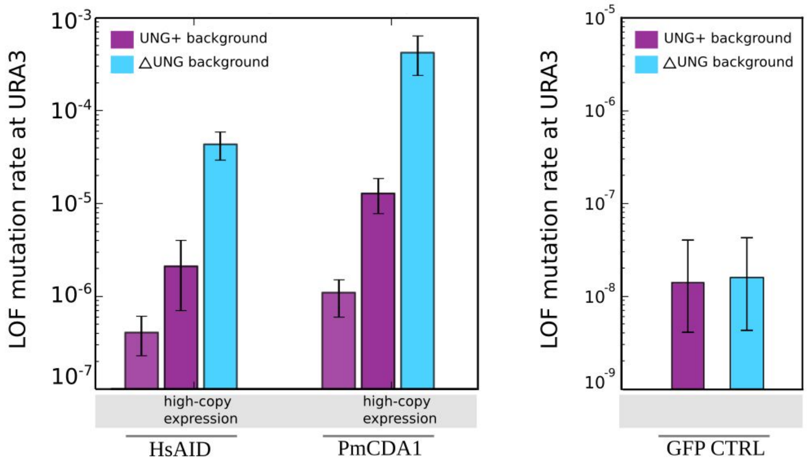

Supplementary Figure 1: Effect of deaminase expression and UNG deletion on untargeted mutation rate at *URA3*. hAID (vectors pCS4329/pCS4330, high/low-copy) and PmCDA1 (vectors pCS4327/pCS4312, high/low-copy), and GFP (pCS1128) were expressed from plasmids in the strain YHM0 with and without UNG disruption. Data indicate *URA3* loss-of-function mutation rate obtained as described in Methods and quantified using the Falcor algorithm. Error bars represent 95% confidence intervals calculated using FALCOR MMS-MLE method measured from 8 independent cultures induced with 0.2% galactose.

Core T7 promoter sequence used in this study

TAATACGACTGACTATAGGGAGA

Core T7 promoter sequence with insertion to generate T7 promoter KO phenotype

TAATACGACTGACCTGGTACAATCGGTACTGGTC**TATAGGGAGA**

Supplementary Figure 2: Schematic of making T7 promoter knockout. YHM0.P_{T7}KO strains were made by disruption of the T7 promoter (bolded) via integration of an intervening 23 bp sequence into strains with function P_{T7}.

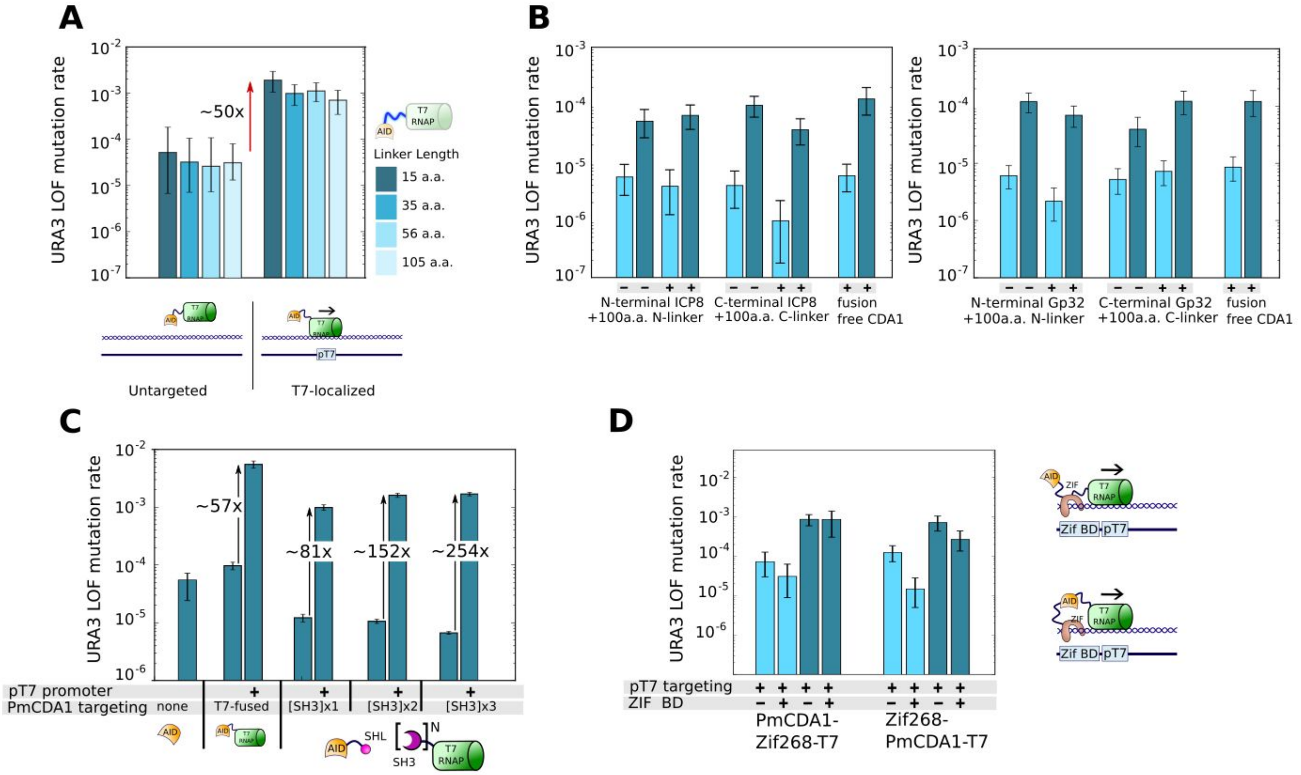

Supplementary Figure 3: Comparison of different strategies to improve the on:off target mutation ratio. Data indicate *URA3* loss-of-function mutation rate obtained as described in Methods and quantified using the Falcor algorithm. Error bars represent 95% confidence intervals calculated using FALCOR MMS-MLE method measured from 8 independent cultures. (a) On and off-target mutation rates in strains YHM0 and YHM0.P_{T7}KO were compared for PmCDA1-T7 RNAP fusions with varying linker lengths. Amino acid linkers of length 5, 35, 56 and 105 correspond to low-copy expression vectors pCS4314, pCS4315, pCS4316, and pCS4317. Samples induced with 0.2% galactose. (b) Untargeted mutation rate of ssDNA binding proteins ICP8 and Gp32 fused to PmCDA1 were compared to determine if ssDNA binding protein - PmCDA1 fusions could increase the non-specific association to DNA, and be used for targeting. N/C terminal fusions of ICP8 to PmCA1 correspond to low-copy expression vectors pCS4321 and pCS4323. N/C terminal fusions of Gp32 to PmCA1 correspond to low-copy expression vectors pCS4320 and pCS4322. Constructs were expressed in YHM0 and induced with 0.2% galactose (dark blue) and 0.02% galactose (light blue). (c) Targeted mutation rate for SHL/SH3 binding domain strategy. SHL was fused to PmCDA1 and SH3 domains to T7 RNAP, both were expressed from a single

low-copy vector. T7 RNAP with one (pCS4324), two (pCS4325) and 3 (pCS4326) SH3 domain fusions were tested. Constructs were expressed in YHM0 and induced with 0.2% galactose. (d) Targeted mutation rate for zinc-finger DNA binding domain targeting strategy. Zif268 was fused to PmCDA1-T7 RNAP as N- (pCS4318) and C-terminal (pCS4319) fusions. Constructs were expressed in YHM0 with addition of a Zif268 binding site upstream of P_{T7} (CSY1167) and induced with 0.2% galactose (dark blue) and 0.02% galactose (light blue).

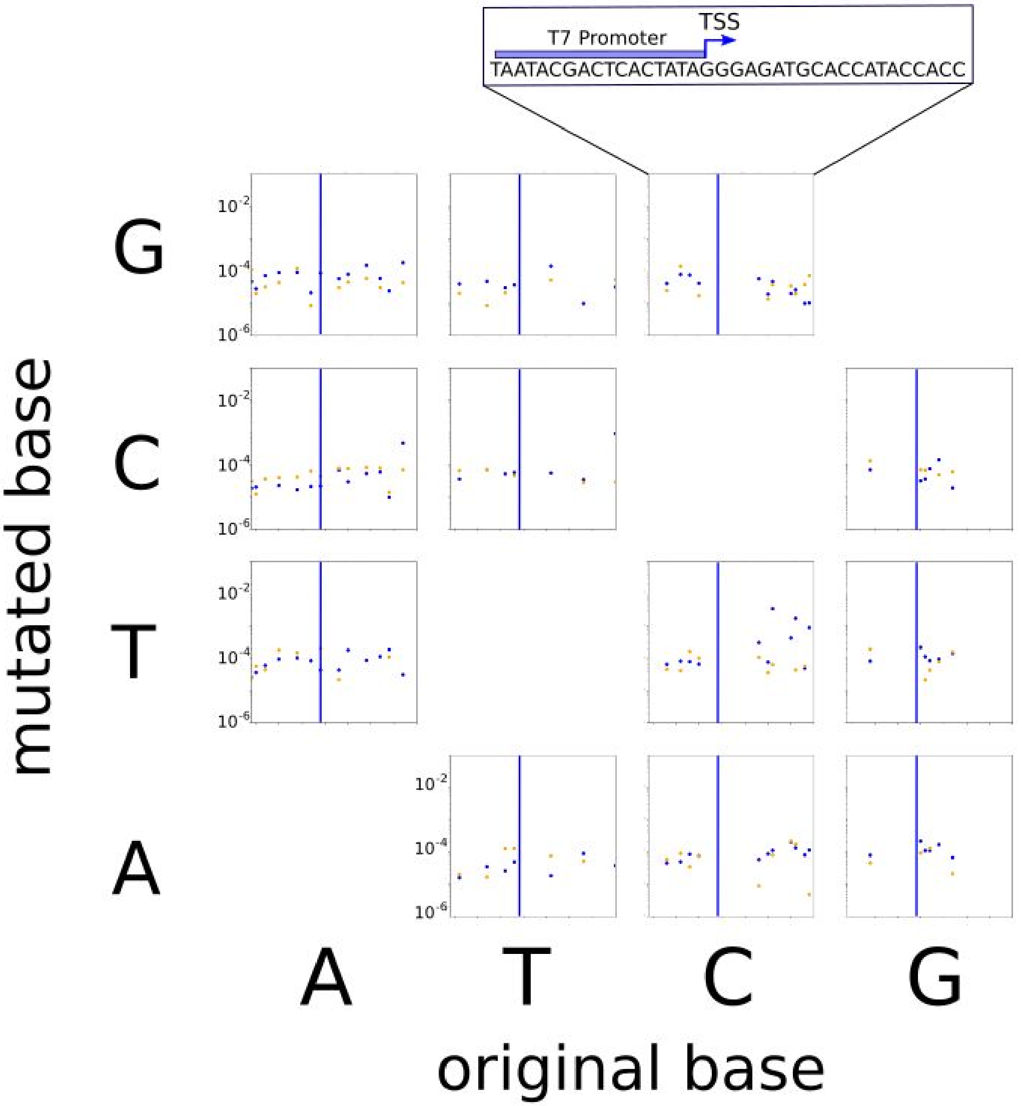

Supplementary Figure 4: Deep sequencing to characterize initiation of mutagenesis and mutagenesis of P_{T7}. Mutation frequencies for all base substitutions plotted by position along the *URA3* sequence for YHM1 (blue) and YHM1.P_{T7}KO (yellow) when induced with 100 nM β-estradiol. Mutations are broken down by substitution type, with the parent sequence along the horizontal (original base) and substitution frequency to a given base along the vertical (mutated base). In order to match YHM1 and YHM1.P_{T7}KO, data from YHM1.P_{T7}KO is off-set to account for the gap created by the 23bp insertion. The P_{T7} promoter comes before the vertical blue bar, and the blue bar represents the transcription start site (TSS). At 12bp from start of transcription

(the third C after the TSS) there is a more than 10-fold increase in C>T mutation compared to the control. Mutation data corresponds to the data in Fig. 1d and was generated by next-generation sequencing at the *URA3* locus as described in Methods.

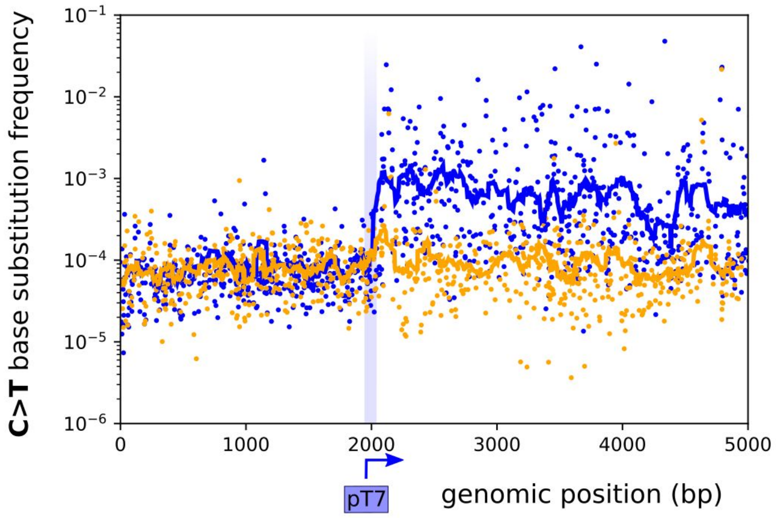

Supplementary Figure 5: Deep sequencing to characterize processivity of PmCDA1-T7 RNAP induced mutagenesis. Mutation frequencies for C>T mutations plotted by position along the *URA3* sequence for YHM1 (blue) and YHM1.P_{T7}KO (yellow) when induced with 100 nM β-estradiol. Sequencing was carried out up to 3,000 bp downstream of P_{T7}. In order to match YHM1 and YHM1.P_{T7}KO, data from YHM1.P_{T7}KO is off-set to account for the gap created by the 23 bp insertion which disrupts P_{T7}.

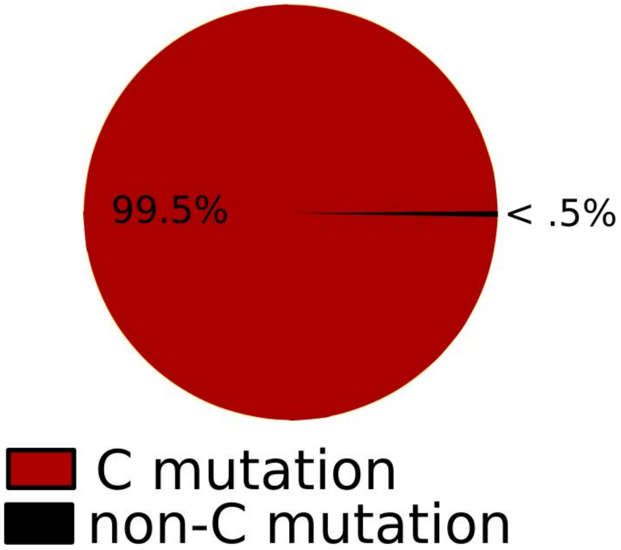

Supplementary Figure 6: Majority of mutations introduced by PmCDA1-T7 RNAP are C>T. Fraction of base substitutions occurring at each base in the mutator strain YHM1 as measured by next-generation-sequencing. Mutation frequencies were calculated from NGS data across bases 1,000-1,200 at *URA3* in YHM1, background sequencing error frequencies (YHM1.P_{T7}KO) were subtracted to account for sequencing errors, and the total substitution frequency at each of A, T, C and G calculated.

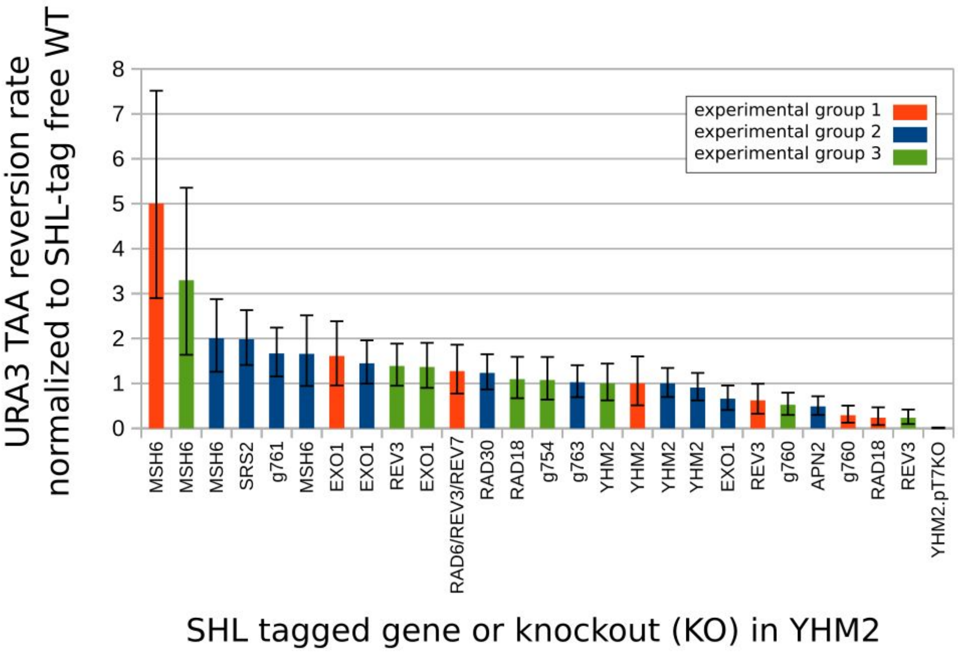

Supplementary Figure 7: Screening of candidate SHL-fusions for increased A/T mutagenesis. A/T targeted mutation rate measured in strain YHM2.TAA featuring different DNA repair proteins targeted by an SHL peptide. A/T rate was measured by gain-of-function fluctuation assay in YHM2.TAA, with reversion of a TAA stop codon in *URA3* and quantified using the Falcor algorithm. Three different replicate experiments were run (colored), normalized to YHM2.TAA, and ordered by fold-increase in TAA reversion rate to highlight the SHL-fuson candidates with the greatest and most consistent A/T rate increase. Error bars represent 95% confidence intervals calculated using FALCOR MMS-MLE method measured from independent cultures induced with 100 nM β-estradiol for up to 24 hours.

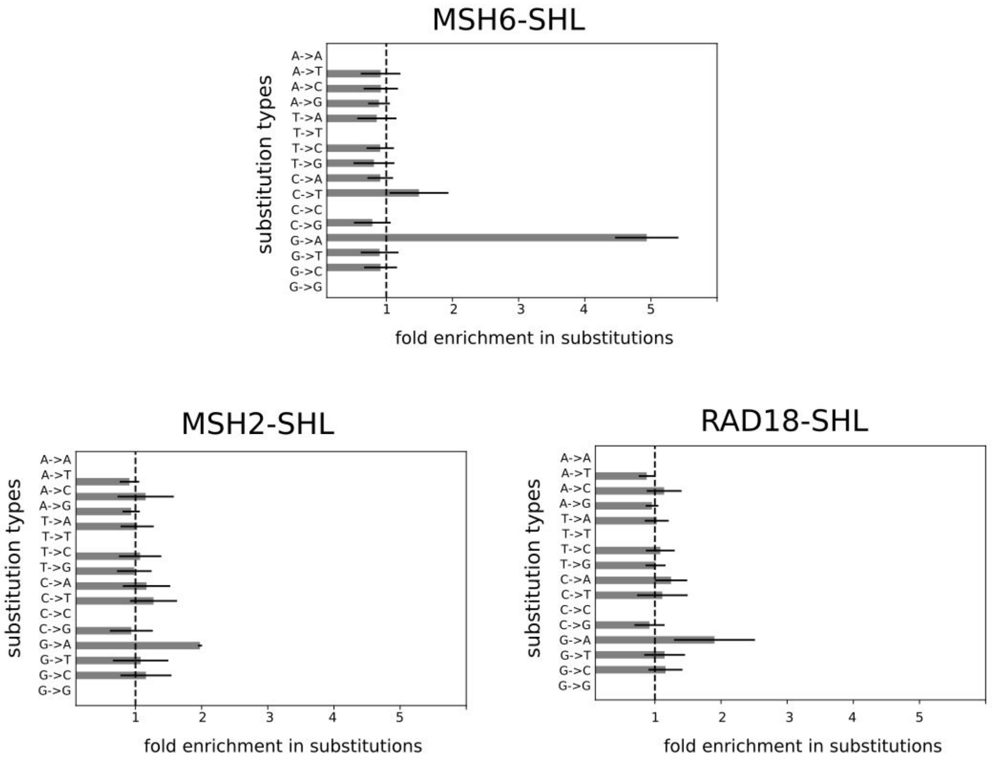

Supplementary Figure 8: Fold increase in substitution rate by substitution type for SHL targeted mismatch repair factors. The ratio of substitution frequencies between YHM2 and YHM2 derived strains with indicated SHL-tags is shown and indicates changes in mutation rate resulting from addition of the SHL-tag. Error bars represent standard deviation calculated across N=600 bps. Ratio of C>T substitution frequency between YHM2 and each SHL-tagged strain is approximately unity, indicating little effect of SHL-tags on C>T substitution frequency. Mutation data generated by next-generation sequencing (NGS) at the *URA3* locus as described in Methods, and cultures were induced with 100 nM β-estradiol for 16 hours. The average substitution frequency was calculated in duplicate for each substitution type between bases 800-1,000 (corresponding to Fig. 1d) and then fold enrichment in substitutions calculated by the ratio of substitution frequencies between YHM2 and indicated SHL-tag variant of YHM2.

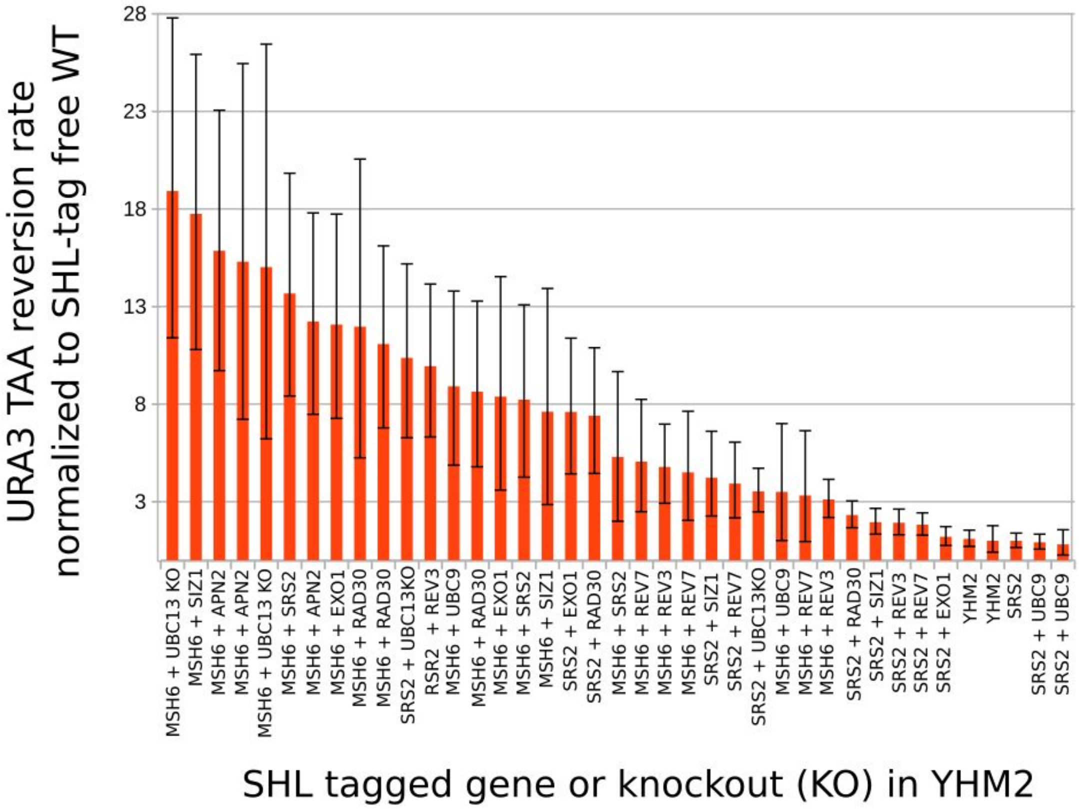

Supplementary Figure 9: Screening of combinatorial SHL-fusions for increased A/T mutagenesis. A/T targeted mutation rate measured in strain YHM2.TAA featuring different deletions or DNA repair proteins targeted by an SHL peptide in pairwise combinations. A/T rate was measured by reversion of a TAA stop codon in *URA3* and quantified using the Falcor algorithm. TAA reversion rate was normalized to YHM2.TAA, and ordered by fold-increase in TAA reversion rate to highlight the SHL-fuson candidates with the greatest and most consistent A/T rate increase for further evaluation. Error bars represent 95% confidence intervals calculated using FALCOR MMS-MLE method measured from 8 independent cultures induced with 100 nM β-estradiol.

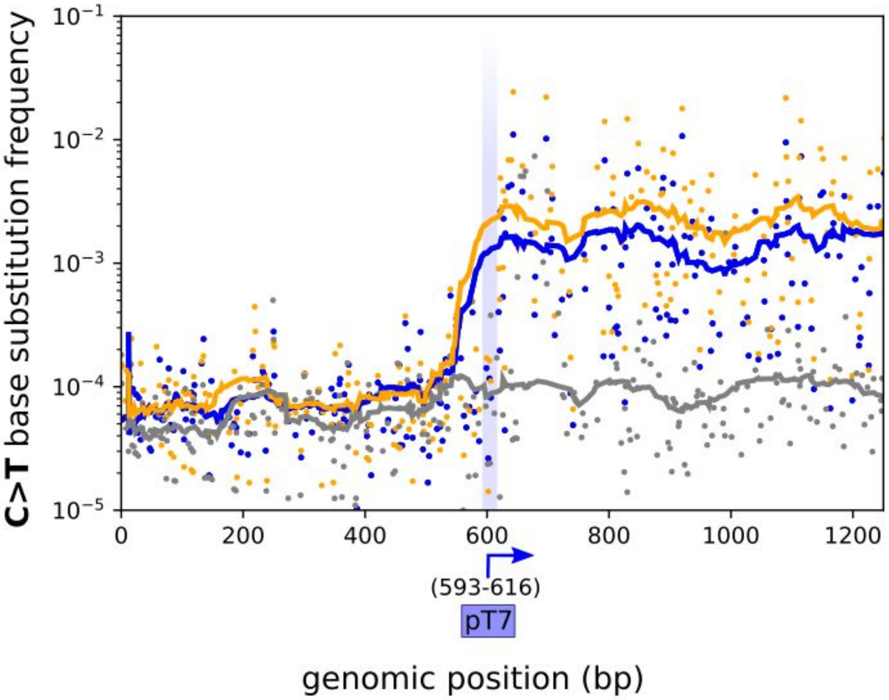

Supplementary Figure 10: C>T mutation is maintained in SHL modified strain YHM4. Mutation frequencies for C>T mutations plotted by position along the *URA3* sequence for YHM4 (blue), YHM1 (yellow) and YHM1.P_{T7}KO (grey) when induced with 100 nM *β*β-estradiol. In order to match YHM4/YHM1 and YHM1.P_{T7}KO, data from YHM1.P_{T7}KO is off-set to account for the gap created by the 23 bp insertion. Mutation data was generated by next-generation sequencing at the *URA3* locus as described in Methods.

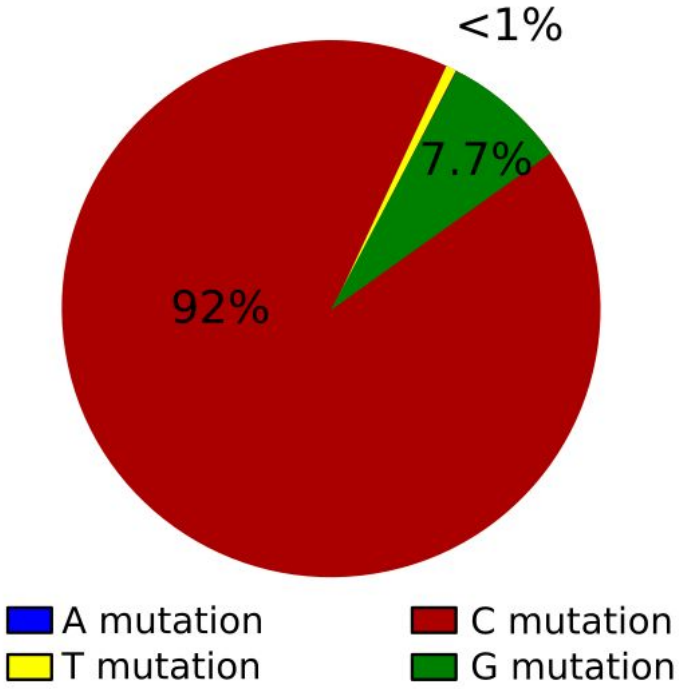

Supplementary Figure 11: Mutations induced in YHM4 are primarily C/G substitutions. Fraction of base substitutions occurring at each base in the mutator strain YHM4 as measured by NGS. Mutation frequencies were calculated in duplicate from NGS data across bases 1,000-1,200 at *URA3* in YHM4 and for each substitution type the background sequencing error frequencies (calculated from YHM2.P_{T7}KO) were subtracted to account for sequencing errors.

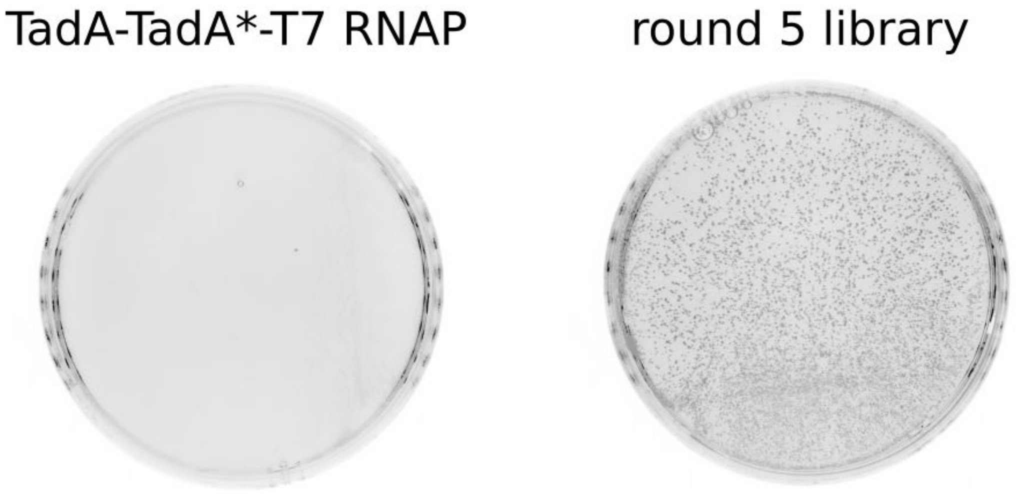

Supplementary Figure 12: Adenosine deaminase engineering yields TadA variants with high TAA reversion rates. CSY1256 containing TadA-TadA*-T7 RNAP (pCS4333) and TadA-Tad*-T7 RNAP variants from round 5 of library selection were induced with galactose for 16 hours and plated on uracil-lacking media. Colony formation indicates reversion of *URA3* containing a premature TAA stop codon to a functional copy of *URA3*.

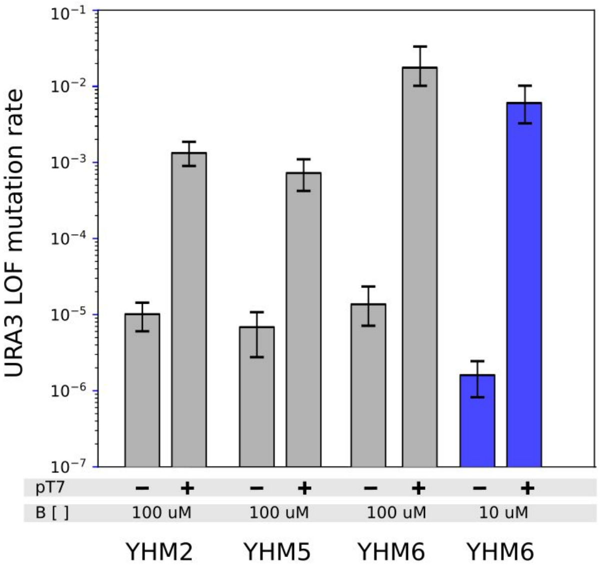

Supplementary Figure 13: TRIDENT strains YHM5 and YHM6 retain similar on and off-target mutation rates to YHM2. Data indicate *URA3* loss-of-function mutation rate obtained as described in Methods and quantified using the Falcor algorithm. Error bars represent 95% confidence intervals calculated using FALCOR MMS-MLE method measured from 80 independent cultures. On and off-target mutation rates in strains YHM2, YHM5, YHM6 and YHM2.P_{T7}KO, YHM5.P_{T7}KO, YHM6.P_{T7}KO. Samples induced with either 100 uM or 10 uM β-estradiol, as noted in the figure.

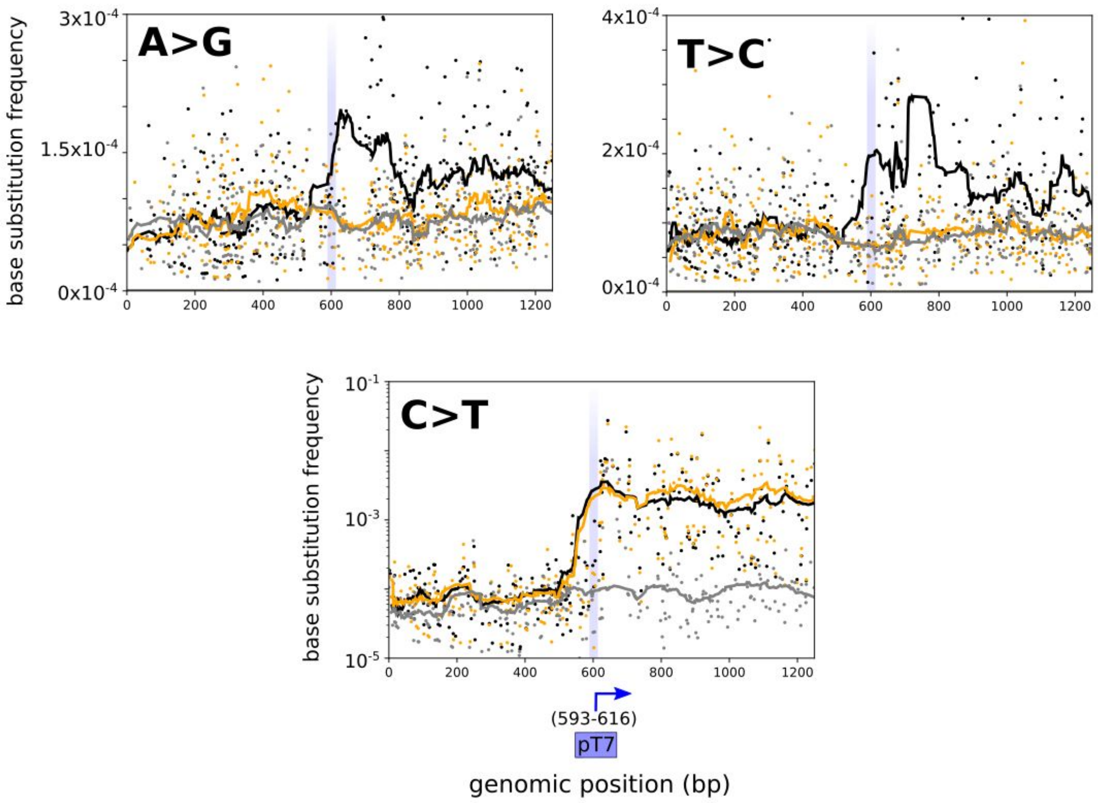

Supplementary Figure 14: TRIDENT induces mutations at all four nucleotides. Mutation frequencies are shown for base substitutions that differ from the control (A>G, T>C, and C>T shown here; G>A in Fig. 3c). Mutation frequencies are plotted by position along the *URA3* sequence for YHM2 (yellow), YHM6 (black), and YHM2.P_{T7}KO (grey) when induced with 100 nM *β*β-estradiol for 16 hours. The blue bar represents the location of a T7 promoter sequence. In order to match YHM6 and YHM2.P_{T7}KO, data from YHM2.P_{T7}KO is off-set to account for the gap created by the 23bp insertion. Mutation data obtained by next-generation sequencing (NGS) at the *URA3* locus as described in Methods.

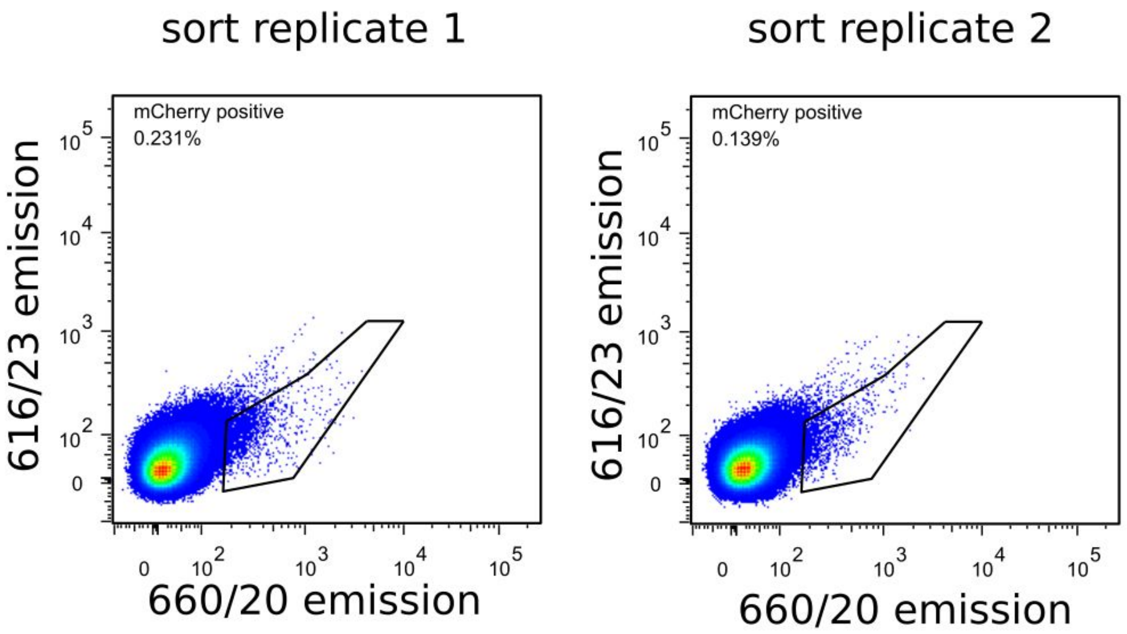

Supplementary Figure 15: TRIDENT can be applied to non-growth-linked traits. A copy of mCherry containing a premature stop codon was expressed in strain YHM6.mCherry.TAA. Mutagenesis was induced with 10 nM β-estradiol for 16 hours and cells were sorted for mCherry-positive fraction of cells according to indicated gates. Due to the particular integration loci (Supplementary Table 1) mCherry signal appears lower than typically seen from plasmid-based expression. mCherry-positive cells were validated for expression by sequencing and re-analysis on a flow cytometer (Fig. 4b).

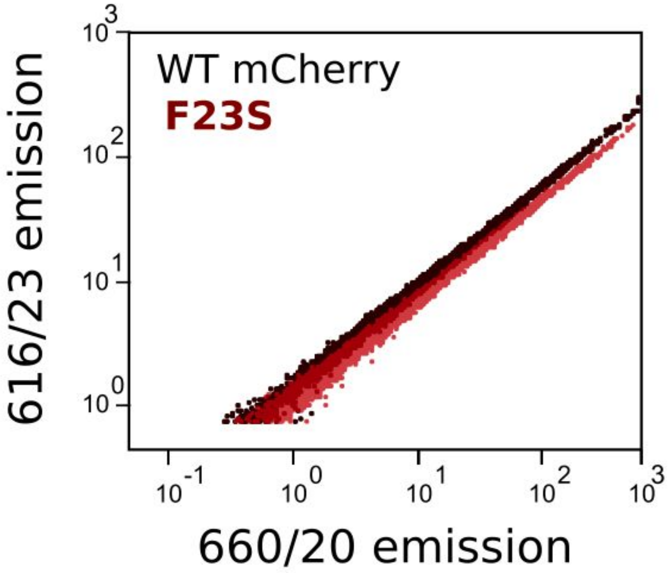

Supplementary Figure 16: TRIDENT can be applied to evolve novel non-growth linked traits. Red-shifted mCherry yeast library analyzed after sort 4 (red) compared to wild-type mCherry expressing population of yeast (black, pCS4335). Cells were grown for 16 hours prior to flow analysis.

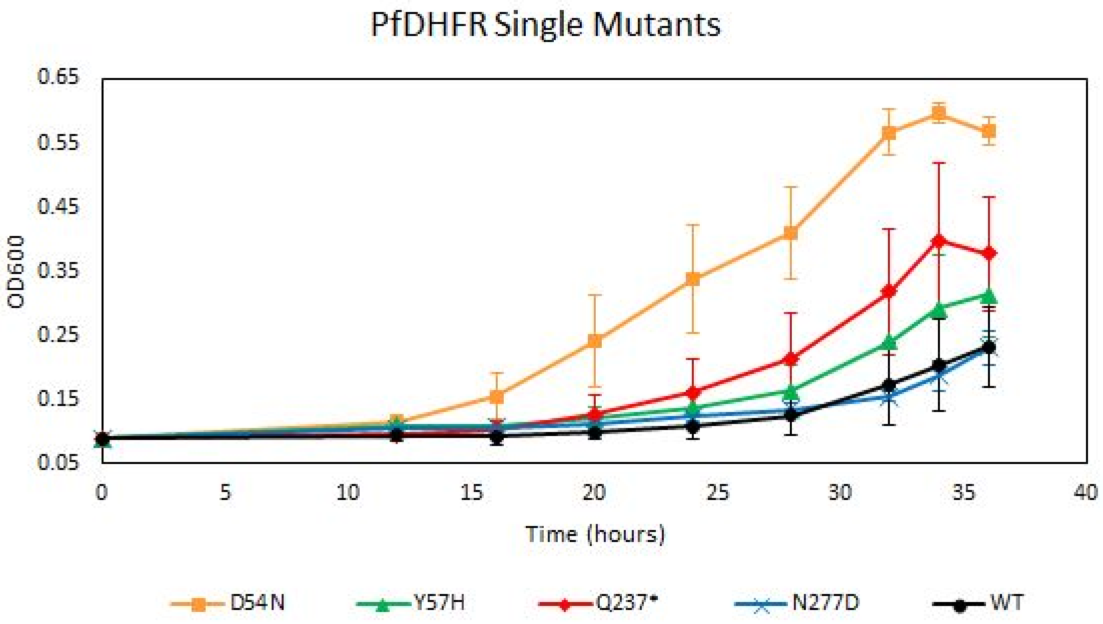

Supplementary Figure 17: Mutations enriched using TRIDENT in the presence of pyrimethamine result in faster growth in media containing pyrimethamine. Mutations found in significant quantities after an 11 day enrichment period including five passages into higher concentrations of pyrimethamine were isolated and tested individually in media containing the maximum concentration of pyrimethamine. Error bars represent the standard deviation of three biological replicates. D54N grew significantly faster than the wild-type enzyme. Y57H and Q237* seemed to grow more quickly than the wild-type, but the difference in optical density was not statistically significant for any timepoint. The N277D variant did not appear to have any impact on the growth rate of the yeast.

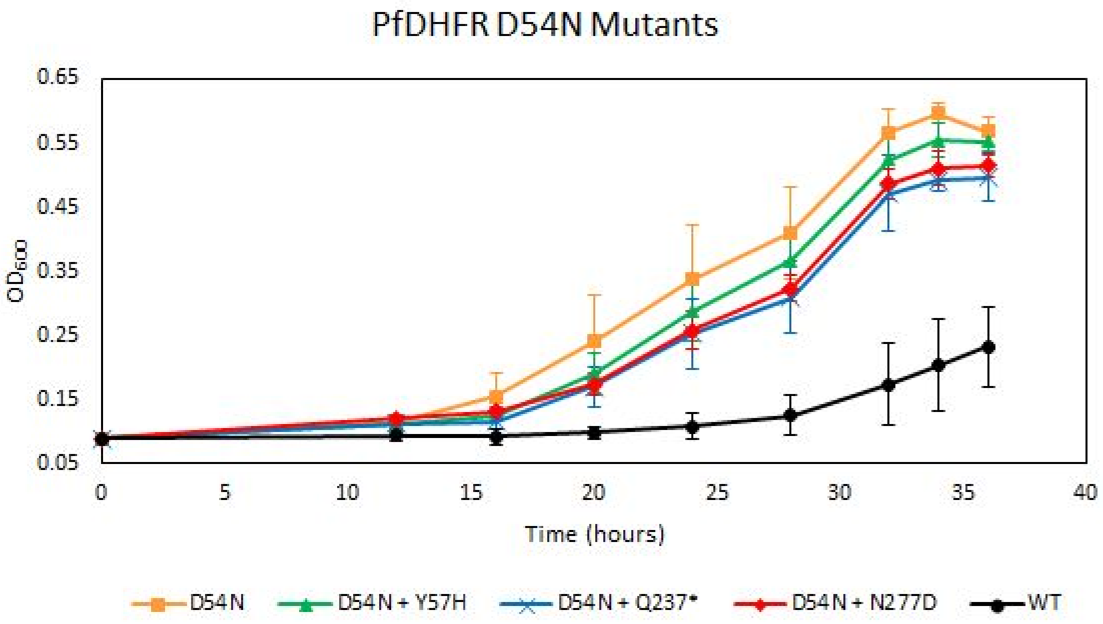

Supplementary Figure 18: Mutations enriched using TRIDENT in the presence of pyrimethamine were tested in combination with D54N to see if they provided additional improvements in growth rate. The less common mutations were tested in combination with D54N, the most common mutation to see if the mutation pairs were beneficial. Error bars represent the standard deviation of three biological replicates. All variants with D54N and an additional mutation resulted in growth that was faster than the wild-type by a statistically significant margin. There was no significant difference in the growth rates between the variants that included the D54N mutation.

Row Identity	Barcode
100 nM Row A	CACATATCAGAGTGCG
100 nM Row B	ACACACAGACTGTGAG
100 nM Row C	ACACATCTCGTGAGAG
100 nM Row D	CACGCACACACGCGCG
100 nM Row E	CACTCGACTCTCGCGT
100 nM Row F	CATATATATCAGCTGT
100 nM Row G	TCTGTATCTCTATGTG
100 nM Row H	ACAGTCGAGCGCTGCG
2 nM Row A	ACACACGCGAGACAGA
2 nM Row B	ACGCGCTATCTCAGAG
2 nM Row C	CTATACGTATATCTAT
2 nM Row D	ACACTAGATCGCGTGT
2 nM Row E	CTCTCGCATACGCGAG
2 nM Row F	CTCACTACGCGCGCGT
2 nM Row G	CGCATGACACGTGTGT
2 nM Row H	CATAGAGAGATAGTAT

Column Identity	Barcode
100 nM Column 1	CACACGCGCGCTATAT
100 nM Column 2	TCACGTGCTCACTGTG
100 nM Column 3	ACACACTCTATCAGAT
100 nM Column 4	CACGACACGACGATGT
100 nM Column 5	CTATACATAGTGATGT
100 nM Column 6	CACTCACGTGTGATAT
100 nM Column 7	CAGAGAGATATCTCTG
100 nM Column 8	CATGTAGAGCAGAGAG
100 nM Column 9	CGCGACACGCTCGCGC
100 nM Column 10	CACAGAGACACGCACA
100 nM Column 11	CTCACACTCTCTCACA
100 nM Column 12	CTCTGCTCTGACTCTC
2 nM Column 1	TATATATGTCTATAGA
2 nM Column 2	TCTCTCTATCGCGCTC
2 nM Column 3	GATGTCTGAGTGTGTG
2 nM Column 4	GAGACTAGAGATAGTG
2 nM Column 5	TCTCGTCGCAGTCTCT
2 nM Column 6	ATGTGTATATAGATAT
2 nM Column 7	GCGCGCGCACTCTCTG
2 nM Column 8	GAGACACGTCGCACAC
2 nM Column 9	ACACATATCGCACTAC
2 nM Column 10	GTGTGTCTCGATGCGC
2 nM Column 11	CGCACACATAGATACA
2 nM Column 12	TGTCATATGAGAGTGT

Supplementary Figure 19: Barcodes used to identify the well of origin during sequencing for PfDHFR mutation experiments. To identify the well of origin with the 180 experimental replicates, front barcodes corresponding to the originating row and back barcodes corresponding to the originating column were added via PCR amplification. “2 nM” and “100 nM” refer to the different β-estradiol concentrations used to induce mutagenesis during growth.

Position	Original BP	Mutated BP	Global % Mutated	Well Count (>25% mutated)	Original Codon	Mutated Codon	Mutation Type	How Found?
42	C	T	2.0	4	I14	I	Silent	Present in >= 4 wells
159	G	A	11.6	26	G53	G	Silent	Global average > 10%
160	G	A	86.5	163	D54	N	Coding	Global average > 85%
169	T	C	15.5	44	Y57	H	Coding	Global average > 15%
426	C	T	8.8	11	I142	I	Silent	Present in >= 4 wells
492	C	T	6.5	5	I164	I	Silent	Present in >= 4 wells
600	C	T	3.1	4	I200	I	Silent	Present in >= 4 wells
624	C	T	2.1	4	I208	I	Silent	Present in >= 4 wells
709	C	T	14.4	50	Q237	*	Coding	Global average > 10%
829	A	G	2.7	5	N277	D	Coding	Present in >= 4 wells

Supplementary Figure 20: Table of significant mutations after PfDHFR mutagenesis. Mutations were considered significant if the global mutation frequency was above 10% or if they were present in at least 4 unique wells.

SUPPLEMENTARY TABLES

Supplementary Table 1: Compilation of integrated strains constructed in this work, with modification and corresponding material request reference number (CSY#).

**Base strains**	**Genotype**	**Source**
**CEN.PK2**	*MAT*a/α *ura3-52/ura3-52 trp1-289/trp1-289 leu2-3_112/leu2-3_112 his3 Δ1/his3 Δ1 MAL2-8C/MAL2-8C SUC2/SUC2*	EUROSCARF acc. No. 30000B
**YHM0**	CEN.PK2 ura3-52-**Δ**,*lyp1::*P_{T7}-P_{URA3}-*URA3*-T_{URA3}, *ung1::ADH1t*	this work
**YHM1**	YHM0 *trp1-289::*P_{Pr3} *-PmCDA1-T7 RNAP*-T_{CYC1}, *leu2-3::ZEV*	this work, ZEV[7]
**YHM2**	YHM0 *trp1-289::*P_{Pr3}*-SH3-SH3-PmCDA1-T7 RNAP*-T_{CYC1}, *leu2-3::ZEV*	this work, ZEV[7]
**YHM3**	YHM2 *msh6***Δ***::SHL-MSH6*	this work
**YHM4**	YHM3 *apn2***Δ***::SHL-APN2*	this work
**YHM5**	YHM2 *his2::*P_{Pr3}*-yeTadA1.0-T7 RNAP*-T_{CYC1}	this work
**YHM6**	YHM4 *his2::*P_{Pr3}*-yeTadA1.0-T7 RNAP*-T_{CYC1}	this work
**CSY1256**	W303 (Matα, ade2–1; ura3–1; his3–11,15; trp1–1; leu2–3,112; can 1– 100) *lyp1::*P_{T7}-P_{URA3}-*URA3*-T_{URA3}, *mag1::ADH1t, ura3-1***Δ***::*HIS3	this work
**CSY1313**	YHM6 lyp1::P_{T7}-P_{TEF}-*PfDHFR*-T_{CYC1}-T_{T7}, *dfr1***Δ**	this work
**CSY1321**	YHM6 lyp1::P_{TEF}-*PfDHFR*-T_{CYC1}-T_{T7}, *dfr1***Δ**	this work
**Derivative strain modifications**		
**.P_{T7}KO**	reference strain *lyp1::*P_{T7}**Δ**::$P_{TAATACGACTGACCTGGTACAATCGGTACTGGTCTATAGGGAGA}$	this work
**.T_{T7}**	reference strain *lyp1::*P_{T7}**Δ**::$P_{TAATACGACTCTATAGGGAGA}$	this work
**.TAA**	reference strain *lyp1::*P_{T7}-P_{URA3}-*URA3*-T_{URA3}**Δ***::*P_{T7}-P_{URA3}-*URA3-E8TAA*-T_{URA3}	this work
**.mcherry**	reference strain + *lyp1::*P_{T7}-P_{URA3}**Δ***::*P_{T7}-$P_{GAL4BS4c1}$-Aga2-mCherry-T_{T7}-T_{AGA1}	this work, GAL4BSC4c1[20]
**.mcherry-TAA**	reference strain + *lyp1::*P_{T7}-P_{URA3}**Δ***::*P_{T7}-$P_{GAL4BS4c1}$-Aga2-mCherry-TAA-T_{T7}-T_{AGA1}	this work
**.SHL-EXO1**	reference strain *exo1***Δ***::SHL-EXO1*	this work

**.SHL-MSH2**	reference strain *msh2Δ::SHL-MSH2*	this work
**.SHL-MSH6**	reference strain *msh6Δ::SHL-MSH6*	this work
**.SHL-RAD18**	reference strain *rad18Δ::RAD18-SHL*	this work
**.SHL-RAD6**	reference strain *rad6Δ::RAD6-SHL*	this work
**.SHL-RAD30**	reference strain *rad30Δ::RAD30-SHL*	this work
**.SHL-REV1**	reference strain *rev1Δ::SHL-REV1*	this work
**.SHL-REV3**	reference strain *rev3Δ::SHL-REV3*	this work
**.SHL-REV7**	reference strain *rev7Δ::SHL-REV7*	this work
**.SHL-APN2**	reference strain *apn2Δ::SHL-APN2*	this work
**.SHL-SRS2**	reference strain *srs2Δ::SRS2-SHL*	this work
**.SHL-SIZ1**	reference strain *siz1Δ::SIZ1-SHL*	this work
**.SHL-UBC9**	reference strain *ubc9Δ::UBC9-SHL*	this work
**.SHL-UBC13**	reference strain *ubc13*-**Δ**	this work
**.SHL-MMS2**	reference strain *mms2*-**Δ**	this work
**.pep-MSH6**	reference strain *msh6Δ::MGSASGSGDS-MSH6*	this work
**.pep-APN2**	reference strain *apn2Δ::MSSSENTGSASGSGD-APN2*	this work

**Parent strains with derivative strain modifications**			
**Strain derivative**	**CSY#**	**Strain derivative**	**CSY#**
**YHM0.P_{T7}KO**	CSY1160	**YHM0 UNG+**	CSY1165
**YHM1.TAA**	CSY1257	**YHM0.zif**	CSY1167
**YHM1.P_{T7}KO**	CSY1258	**YHM2.SHL-EXO1**	CSY1199
**YHM2.TAA**	CSY1259	**YHM2.SHL-RAD18**	CSY1201
**YHM2.P_{T7}KO**	CSY1194	**yA4 UNG+**	CSY1262

**YHM3.TAA**	CSY1260		**yA4 UNG-**	CSY1263
**YHM3.P$_{T7}$KO**	CSY1261		**YHM2.TAA.SHL-REV3**	CSY1271
**YHM2.T$_{T7}$**	CSY1283		**YHM2.TAA.SHL-REV7**	CSY1272
**YHM2.TAA.SHL-EXO1**	CSY1264		**YHM2.TAA.SHL-APN2**	CSY1273
**YHM2.TAA.SHL-MSH2**	CSY1256		**YHM2.TAA.SHL-SRS2**	CSY1274
**YHM2.TAA.SHL-MSH6**	CSY1260		**YHM2.TAA.SHL-SIZ1**	CSY1275
**YHM2.TAA.SHL-RAD18**	CSY1267		**YHM2.TAA.SHL-UBC9**	CSY1276
**YHM2.TAA.SHL-RAD6**	CSY1268		**YHM2.TAA.SHL-UBC13**	CSY1277
**YHM2.TAA.SHL-RAD30**	CSY1269		**YHM2.TAA.SHL-MMS2**	CSY1278
**YHM2.TAA.SHL-REV1**	CSY1270		**YHM2.TAA.pep-MSH6**	CSY1279
**YHM2.TAA.SHL-REV3**	CSY1271		**YHM2.TAA.pep-APN2.MSH6**	CSY1281
**YHM2.TAA.SHL-REV7**	CSY1272		**YHM2.TAA.pep-APN2**	CSY1280
**YHM2.TAA.SHL-APN2**	CSY1273		**YHM2.TAA.SHL-MMS2**	CSY1278
**YHM2.TAA.SHL-SRS2**	CSY1274		**YHM2.TAA.pep-MSH6**	CSY1279
**YHM2.TAA.SHL-SIZ1**	CSY1275		**YHM2.TAA.pep-APN2**	CSY1280
**YHM2.TAA.SHL-UBC9**	CSY1276		**YHM2.TAA.pep-APN2.MSH6**	CSY1281
**YHM2.TAA.SHL-UBC13**	CSY1277		**YHM6**	CSY1255
**YHM5**	CSY1254			

Supplementary Table 2: List of genes implicated in B-cell somatic hypermutation mutation diversity, and corresponding yeast homologue(s).

**B-cell gene**	**Yeast homologue**	**Function**	**Source**
EXO1	EXO1	Exonuclease	*Altered somatic hypermutation and reduced class-switch recombination in exonuclease 1-mutant mice.*
MSH2	MSH2	MMR and error prone repair	*Somatic hypermutation in MutS homologue (MSH)3-, MSH6-, and MSH3/MSH6-deficient mice reveals a role for*

			the MSH2-MSH6 heterodimer in modulating the base substitution pattern
MSH6	MSH6	MMR and error prone repair	*Somatic hypermutation in MutS homologue (MSH)3-, MSH6-, and MSH3/MSH6-deficient mice reveals a role for the MSH2-MSH6 heterodimer in modulating the base substitution pattern*
RAD18	RAD18	MMR / PCNA ubiquitination for TLS repair	*Yeast DNA repair proteins Rad6 and Rad18 form a heterodimer that has ubiquitin conjugating, DNA binding, and ATP hydrolytic activities*
RAD6	RAD6	TLS regulator / PCNA ubiquitination for TLS repair	*Yeast DNA repair proteins Rad6 and Rad18 form a heterodimer that has ubiquitin conjugating, DNA binding, and ATP hydrolytic activities*
Pol η	RAD30	TLS polymerase	*DNA polymerase η is an A-T mutator in somatic hypermutation of immunoglobulin variable genes*
REV1	REV1	TLS polymerase	*A Critical Role for REV1 in Regulating the Induction of C:G Transitions and A:T Mutations during Ig Gene Hypermutation*
Pol ζ	REV3	TLS polymerase subunit	*Altered Ig Hypermutation Pattern and Frequency in Complementary Mouse Models of DNA Polymerase ζ Activity*
Pol ζ	REV7	TLS polymerase subunit	*Altered Ig Hypermutation Pattern and Frequency in Complementary Mouse Models of DNA Polymerase ζ Activity*
APE1/2	APN1/2	Apurinic endonuclease key to formation of ssDNA gaps	*Differential expression of APE1 and APE2 in germinal centers promotes error-prone repair and A: T mutations during somatic hypermutation*
	SRS2	Inhibitor of recombination repair	*SUMO-modified PCNA recruits Srs2 to prevent recombination during S phase*
	SIZ1	Regulation of PCNA ubiquitination and SUMOylation	*RAD6-dependent DNA repair is linked to modification of PCNA by ubiquitin and SUMO, SUMO-modified PCNA recruits Srs2 to prevent recombination during S phase*

UBE2I	UBC9	Regulation of PCNA ubiquitination	*RAD6-dependent DNA repair is linked to modification of PCNA by ubiquitin and SUMO, Somatic hypermutation of immunoglobulin genes: lessons from proliferating cell nuclear antigenK164R mutant mice*
UBE2N	UBC13	Error-free lesion bypass protein	*Avoidance of APOBEC3B-induced mutation by error-free lesion bypass, Somatic hypermutation of immunoglobulin genes: lessons from proliferating cell nuclear antigenK164R mutant mice*
UBE2V2	MMS2	Error-free lesion bypass protein	*Avoidance of APOBEC3B-induced mutation by error-free lesion bypass, Somatic hypermutation of immunoglobulin genes: lessons from proliferating cell nuclear antigenK164R mutant mice*

Supplementary Table 3: Growth rates of various TRIDENT yeast strains compared to wild-type CEN.PK2. Samples were measured in triplicate during exponential phase growth. No mean had a statistically significant difference from CEN.PK growth at $p<0.05$ threshold (one sided t-test).

**Strain**	**β-estradiol concentration**	**Mean growth rate (1/hours)**	**Standard deviation (1/hours)**
	**5nM**	0.105	0.0027
	**100nM**	0.104	0.0014
**YHM2**	**0nM**	0.105	0.0008
	**5nM**	0.105	0.0024
	**100nM**	0.094	0.0030
**YHM5**	**0nM**	0.105	0.0004
	**5nM**	0.094	0.0046
	**100nM**	0.083	0.0055
**YHM6**	**0nM**	0.096	0.0028
	**5nM**	0.101	0.0010
	**100nM**	0.101	0.0018
**CEN.PK**	**0nM**	0.101	0.0024

**Supplementary Table 4:** Plasmid cassettes and gRNA sequences used in this study.		
**Plasmid**	**Description**	**Source**
pCS4312	CEN/ARS vector, P_{GAL1}-PmCDA1-T_{CYC1}, TRP1 selectable marker	this work
pCS4313	CEN/ARS vector, P_{GAL1}T7 RNAP-T_{CYC1}, TRP1 selectable marker	this work
pCS4314	CEN/ARS vector, P_{GAL1}-PmCDA1-8AA linker-T7 RNAP-T_{CYC1}, TRP1 selectable marker	this work
pCS4315	CEN/ARS vector, P_{GAL1}-PmCDA1-100AA linker-T7 RNAP-T_{CYC1}, TRP1 selectable marker	this work
pCS4316	CEN/ARS vector, P_{GAL1}-PmCDA1-50AA linker-T7 RNAP-T_{CYC1}, TRP1 selectable marker	this work
pCS4317	CEN/ARS vector, P_{GAL1}-PmCDA1-30AA linker-T7 RNAP-T_{CYC1}, TRP1 selectable marker	this work
pCS4318	CEN/ARS vector, P_{GAL1} - Zif268-PmCDA1-8AA linker-T7 RNAP -T_{CYC1}, TRP1 selectable marker	this work
pCS4319	CEN/ARS vector, P_{GAL1} - PmCDA1-100AA linker -Zif268-80AA linker-T7 RNAP-T_{CYC1}, TRP1 selectable marker	this work
pCS4320	CEN/ARS vector, P_{GAL1}-PmCDA1-100AA linker -Gp32-T_{CYC1}, TRP1 selectable marker	this work
pCS4321	CEN/ARS vector, P_{GAL1}-PmCDA1-100AA linker -ICP8-T_{CYC1}, TRP1 selectable marker	this work
pCS4322	CEN/ARS vector, P_{GAL1}-Gp32-100AA linker-PmCDA1-T_{CYC1}, TRP1 selectable marker	this work
pCS4323	CEN/ARS vector, P_{GAL1}-ICP8-100AA linker-PmCDA1-T_{CYC1}, TRP1 selectable marker	this work
pCS4324	CEN/ARS vector, P_{GAL10}-PmCDA1-SHL-T_{ADH1}, P_{GAL10}-PmCDA1-T_{CYC1} P_{GAL1}-SH3-30AA linker-T7 RNAP-T_{CYC1}, TRP1 selectable marker	this work
pCS4325	CEN/ARS vector, P_{GAL10}-PmCDA1-SHL-T_{ADH1}, P_{GAL1}-SH3-SH3-30AA linker-T7 RNAP-T_{CYC1}, TRP1 selectable marker	this work
pCS4326	CEN/ARS vector, P_{GAL10}-PmCDA1-SHL-T_{ADH1}, P_{GAL1}-SH3-SH3-SH3-30AA linker-T7 RNAP-T_{CYC1}, TRP1 selectable marker	this work

pCS4327	2μ vector, P_{GAL10}-PmCDA1-T_{ADH1}, LEU2 selectable marker	addgene 60818
pCS1128	CEN/ARS vector, P_{GAL1}-GFP-T_{CYC1}, TRP1 selectable marker	this work
pCS4329	2μ vector, P_{GAL1}-PmCDA1-T_{CYC1}, LEU2 selectable marker	addgene 60810
pCS4330	CEN/ARS vector, P_{GAL1}-hAID-T_{CYC1}, TRP1 selectable marker	this work
pCS4331	CEN/ARS vector, P_{GAL1}-TadA*-100AA linker-T7 RNAP-T_{CYC1}, TRP1 selectable marker	this work
pCS4332	CEN/ARS vector, P_{GAL1}-ABE7.10-100AA linker-T7 RNAP-T_{CYC1}, TRP1 selectable marker	this work
pCS4333	CEN/ARS vector, P_{GAL1}-yeTadA1.0-100AA linker-T7 RNAP-T_{CYC1}, TRP1 selectable marker	this work<br>addgene 137735
pCS3306	pET28a vector	addgene 60733
pCS4334	CEN/ARS vector, P_{TEF1}-mCherry-T_{ADH1}, URA3 selectable marker	this work
pCS4335	CEN/ARS vector, P_{T7}-P_{TEF1}-mCherry-T_{ADH1}, URA3 selectable marker	this work
pCS4336	CEN/ARS vector, P_{T7}-P_{TEF1}-mCherry-F23S-T_{ADH1}, URA3 selectable marker	this work
pCS4337	F1 ori vector, P_{T7}-mCherry-T_{T7}, KAN selectable marker	this work
pCS4338	F1 ori vector, P_{T7}-mCherry-F23S-T_{T7}, KAN selectable marker	this work
pCS4601	CEN/ARS vector, P_{TEF1}-PfDHFR-T_{CYC1}-T_{T7}, TRP1 selectable marker	this work

Supplementary Table 4 cont.: gRNAs used in this study; all gRNAs are expressed from pCAS as described in the Methods section.

Plasmid	Description	Source
g479	EXO1	TAGAAAGGAATGGGTATCCA
g480	MSH2	TTAAAAGTATGTCCTCCACT
g481	MSH6	AGGGGTAGCTGGGGCCATTT
g482	RAD18	CGCTTGCAGTGGTTATTTGG
g669	RAD6	AGGAGACGGTAGAGAAATCT

g680	RAD30	AAAAAATGATAAGATGTTTT
g484	REV1	TTCTAGGCATATCCAGCGAT
g663	REV3	CGACACAATACAGAGCGATA
g666	REV7	TGAATAGATGGGTAGAGAAG
g642	APN2	ATCAAGCGAAAACACGTTAC
g842	SRS2	AAAAAGTCAAAATTAAACAA
g760	SIZ1	TATGGAAAGAAATACAACAG
g761	UBC9	CTAAACAGTACTCTAAATAG
g763	UBC13	ATTACCCAAGAGAATAATCA
g754/755	MMS2	CTATCCAGATTCTCCCCCAA
g332	UNG1	TATCAAGACAGAGACAAAGG
pCS3700	URA3	CCTTCGTTCTTCCTTCTGCT
pCS3701	LEU2	GGTAAGAGAAAGGAAGACGA
pCS3702	TRP1	TTTTCGACCGAATTCTTAAT
pCS3703	HIS2	TCTATCAACTCAAATGATAC
pCS3288	LYP1	CATAATAACGTCCAATAAAT
g834	URA3 (for TAA)	CATGTCGAAAGCTACATATA
g796	$P_{T7\text{-}URA3}$	TCGGGGCTGGCTTAACTATG
g797	$P_{T7\text{-}URA3}$	GATGAATTGAAAAGGTGGTA
g865	mCherry (for TAA)	GGCTAGCGAAATAATGTCTA
OKJ 581	DFR1	GAAAGAATCTACGTGATTGG

**Supplementary Table 5:** Protein sequences used in this study	
**Name**	**Sequence**

SH3-**SH3-**30AA linker**-T7 RNAP**	MPKKKRKVGGGGSGGGGSAEYVRALFDFNGNDEEDLPFKKGDILRIRDKPEEQWWNAEDSEGKRGMIPVPYVEKYSGDYKDHDGDYKDHDIDYKDDDDKSRGSGSM**PKKKRKVGGGGSGGGGSAEYVRALFDFNGNDEEDLPFKKGDILRIRDKPEEQWWNAEDSEGKRGMIPVPYVEKYSGDYKDHDGDYKDHDIDYKDDDDKSRGSG**GAEYVRALFDFNGNDEEDLPFKKGDILRIRDNQ**VNTINIAKNDFSDIELAAIPFNTLADHYGERLAREQLALEHESYEMGEARFRKMFERQLKAGEVADNAAAKPLITTLLPKMIARINDWFEEVKAKRGKRPTAFQFLQEIKPEAVAYITIKTTLACLTSADNTTVQAVASAIGRAIEDEARFGRIRDLEAKHFKKNVEEQLNKRVGHVYKKAFMQVVEADMLSKGLLGGEAWSSWHKEDSIHVGVRCIEMLIESTGMVSLHRQNAGVVGQDSETIELAPEYAEAIATRAGALAGISPMFQPCVVPPKPWTGITGGGYWANGRRPLALVRTHSKKALMRYEDVYMPEVYKAINIAQNTAWKINKKVLAVANVITKWKHCPVEDIPAIEREELPMKPEDIDMNPEALTAWKRAAAAVYRKDKARKSRRISLEFMLEQANKFANHKAIWFPYNMDWRGRVYAVSMFNPQGNDMTKGLLTLAKGKPIGKEGYYWLKIHGANCAGVDKVPFPERIKFIEENHENIMACAKSPLENTWWAEQDSPFCFLAFCFEYAGVQHHGLSYNCSLPLAFDGSCSGIQHFSAMLRDEVGGRAVNLLPSETVQDIYGIVAKKVNEILQADAINGTDNEVVTVTDENTGEISEKVKLGTKALAGQWLAYGVTRSVTKRSVMTLAYGSKEFGFRQQVLEDTIQPAIDSGKGLMFTQPNQAAGYMAKLIWESVSVTVVAAVEAMNWLKSAAKLLAAEVKDKKTGEILRKRCAVHWVTPDGFPVWQEYKKPIQTRLNLMFLGQFRLQPTINTNKDSEIDAHKQESGIAPNFVHSQDGSHLRKTVVWAHEKYGIESFALIHDSFGTIPADAANLFKAVRETMVDTYESCDVLADFYDQFADQLHESQLDKMPALPAKGNLNLRDILESDFAFA***
PmCDA1-**SHL**	MVAWSHPQFEKGGGSMTDAEYVRIHEKLDIYTFKKQFFNNKKSVSHRCYVLFELKRRGERRACFWGYAVNKPQSGTERGIHAEIFSIRKVEEYLRDNPGQFTINWYSSWSPCADCAEKILEWYNQELRGNGHTLKIWACKLYYEKNARNQIGLWNLRDNGVGLNVMVSEHYQCCRKIFIQSSHNQLNENRWLEKTLKRAEKRRSELSIMIQVKILHTTKSPAVSSMVD**PPPALPPKRRREFGPGTSFCSL***
BE7.10 (heteromer of TadA-TadA*)	MSEVEFSHEYWMRHALTLAKRAWDEREVPVGAVLVHNNRVIGEGWNRPIGRHDPTAHAEIMALRQGGLVMQNYRLIDATLYVTLEPCVMCAGAMIHSRIGRVVFGARDAKTGAAGSLMDVLHHPGMNHRVEITEGILADECAALLSDFFRMRRQEIKAQKKAQSSTDSGGSSGGSSGSETPGTSESATPESSGGSSGGSSEVEFSHEYWMRHALTLAKRARDEREVPVGAVLVLNNRVIGEGWNRAIGLHDPTAHAEIMALRQGGLVMQNYRLIDATLYVTFEPCVMCAGAMIHSRIGRVVFGVRNAKTGAAGSLMDVLHYPGMNHRVEITEGILADECAALLCYFFRMPRQVFNAQKKAQSSTD*
yeTadA1.0 (monomer)	MSEVEFSHEYWMRHALTLAKRARDEREVPVGAVLVLNNRVIGEGWNRAIGLHDPTAHAEIMALRQGGLVMQNYRLIDATLYVTFEPCVMCAGAMIHSRIGRVVFGVRNAKTGAAGSLMDVLHYPGMNHSVEITEGILADECAALLCYFFRMPRRVFNAQKKAQPSTG*
**yeTadA1.0**-100	**MSEVEFSHEYWMRHALTLAKRARDEREVPVGAVLVLNNRVIGEGWNRAIGLH**

AA linker-T7 RNAP	**DPTAHAEIMALRQGGLVMQNYRLIDATLYVTFEPCVMCAGAMIHSRIGRVVF**<br>**GVRNAKTGAAGSLMDVLHYPGMNHSVEITEGILADECAALLCYFFRMPRRVF**<br>**NAQKKAQPSTG**SGGGGSAEYVRALFDFNGNDEEDLPFKKGDILRIRDKPEEQWWN<br>AEDSEGKRGMIPVPYVEKYSGDYKDHDGDYKDHDIDYKDDDNQVNTINIAKNDFS<br>DIELAAIPFNTLADHYGERLAREQLALEHESYEMGEARFRKMFERQLKAGEVADNA<br>AAKPLITTLLPKMIARINDWFEEVKAKRGKRPTAFQFLQEIKPEAVAYITIKTTLACL<br>TSADNTTVQAVASAIGRAIEDEARFGRIRDLEAKHFKKNVEEQLNKRVGHVYKKAF<br>MQVVEADMLSKGLLGGEAWSSWHKEDSIHVGVRCIEMLIESTGMVSLHRQNAGV<br>VGQDSETIELAPEYAEAIATRAGALAGISPMFQPCVVPPKPWTGITGGGYWANGRR<br>PLALVRTHSKKALMRYEDVYMPEVYKAINIAQNTAWKINKKVLAVANVITKWKH<br>CPVEDIPAIEREELPMKPEDIDMNPEALTAWKRAAAAVYRKDKARKSRRISLEFML<br>EQANKFANHKAIWFPYNMDWRGRVYAVSMFNPQGNDMTKGLLTLAKGKPIGKEG<br>YYWLKIHGANCAGVDKVPFPERIKFIEENHENIMACAKSPLENTWWAEQDSPFCFL<br>AFCFEYAGVQHHGLSYNCSLPLAFDGSCSGIQHFSAMLRDEVGGRAVNLLPSETVQ<br>DIYGIVAKKVNEILQADAINGTDNEVVTVTDENTGEISEKVKLGTKALAGQWLAYG<br>VTRSVTKRSVMTLAYGSKEFGFRQQVLEDTIQPAIDSGKGLMFTQPNQAAGYMAK<br>LIWESVSVTVVAAVEAMNWLKSAAKLLAAEVKDKKTGEILRKRCAVHWVTPDGF<br>PVWQEYKKPIQTRLNLMFLGQFRLQPTINTNKDSEIDAHKQESGIAPNFVHSQDGSH<br>LRKTVVWAHEKYGIESFALIHDSFGTIPADAANLFKAVRETMVDTYESCDVLADFY<br>DQFADQLHESQLDKMPALPAKGNLNLRDILESDFAFA*
**SHL**-Msh6p	M**PPPALPPKRRREFGPGTSFCSL**MAPATPKTSKTAHFENGSTSSQKKMKQSSLLSF<br>FSKQVPSGTPSKKVQKPTPATLENTATDKITKNPQGGKTGKLFVDVDEDNDLTIAEE<br>TVSTVRSDIMHSQEPQSDTMLNSNTTEPKSTTTDEDLSSSQSRRNHKRRVNYAESDD<br>DDSDTTFTAKRKKGKVVDSESDEDEYLPDKNDGDEDDDIADDKEDIKGELAEDSG<br>DDDDLISLAETTSKKKFSYNTSHSSSPFTRNISRDNSKKKSRPNQAPSRSYNPSHSQP<br>SATSKSSKFNKQNEERYQWLVDERDAQRRPKSDPEYDPRTLYIPSSAWNKFTPFEK<br>QYWEIKSKMWDCIVFFKKGKFFELYEKDALLANALFDLKIAGGGRANMQLAGIPE<br>MSFEYWAAQFIQMGYKVAKVDQRESMLAKEMREGSKGIVKRELQCILTSGTLTDG<br>DMLHSDLATFCLAIREEPGNFYNETQLDSSTIVQKLNTKIFGAAFIDTATGELQMLEF<br>EDDSECTKLDTLMSQVRPMEVVMERNNLSTLANKIVKFNSAPNAIFNEVKAGEEFY<br>DCDKTYAEIISSEYFSTEEDWPEVLKSYYDTGKKVGFSAFGGLLYYLKWLKLDKNL<br>ISMKNIKEYDFVKSQHSMVLDGITLQNLEIFSNSFDGSDKGTLFKLFNRAITPMGKR<br>MMKKWLMHPLLRKNDIESRLDSVDSLLQDITLREQLEITFSKLPDLERMLARIHSRT<br>IKVKDFEKVITAFETIIELQDSLKNNDLKGDVSKYISSFPEGLVEAVKSWTNAFERQK<br>AINENIIVPQRGFDIEFDKSMDRIQELEDELMEILMTYRKQFKCSNIQYKDSGKEIYTI<br>EIPISATKNVPSNWVQMAANKTYKRYYSDEVRALARSMAEAKEIHKTLEEDLKNR<br>LCQKFDAHYNTIWMPTIQAISNIDCLLAITRTSEYLGAPSCRPTIVDEVDSKTNTQLN<br>GFLKFKSLRHPCFNLGATTAKDFIPNDIELGKEQPRLGLLTGANAAGKSTILRMACI<br>AVIMAQMGCYVPCESAVLTPIDRIMTRLGANDNIMQGKSTFFVELAETKKILDMAT<br>NRSLLVVDELGRGGSSSDGFAIAESVLHHVATHIQSLGFFATHYGTLASSFKHHPQV<br>RPLKMSILVDEATRNVTFLYKMLEGQSEGSFGMHVASMCGISKEIIDNAQIAADNLE<br>HTSRLVKERDLAANNLNGEVVSVPGGLQSDFVRIAYGDGLKNTKLGSGEGVLNYD<br>WNIKRNVLKSLFSIIDDLQS*

**SHL**-Apn2p	MSSSENT**SSMVDPPPALPPKRRREFGPGT**LLDGKSENTIRFLTFNVNGIRTFFHYQP FSQMNQSLRSVFDFFRADIITFQELKTEKLSISKWGRVDGFYSFISIPQTRKGYSGVG CWIRIPEKNHPLYHALQVVKAEEGITGYLTIKNGKHSAISYRNDVNQGIGGYDSLDP DLDEKSALELDSEGRCVMVELACGIVIISVYCPANSNSSEEGEMFRLRFLKVLLRRV RNLDKIGKKIVLMGDVNVCRDLIDSADTLEQFSIPITDPMGGTKLEAQYRDKAIQFII NPDTPHRRIFNQILADSLLPDASKRGILIDTTRLIQTRNRLKMYTVWNMLKNLRPSN YGSRIDFILVSLKLERCIKAADILPDILGSDHCPVYSDLDILDDRIEPGTTQVPIPKFEA RYKYNLRNHNVLEMFAKKDTNKESNKQKYCVSKVMNTKKNSNIKNKSLDSFFQK VNGEKDDRIKESSEIPQQAKKRISTPKLNFKDVFGKPPLCRHGEESMLKTSKTSANP GRKFWICKRSRGDSNNTESSCGFFQWV*
**random-linker**-Msh6p	M**GSASGSGD**SMAPATPKTSKTAHFENGSTSSQKKMKQSSLLSFFSKQVPSGTPSKK VQKPTPATLENTATDKITKNPQGGKTGKLFVDVDEDNDLTIAEETVSTVRSDIMHS QEPQSDTMLNSNTTEPKSTTTDEDLSSSQSRRNHKRRVNYAESDDDDSDTTFTAKR KKGKVVDSESDEDEYLPDKNDGDEDDDIADDKEDIKGELAEDSGDDDDLISLAETT SKKKFSYNTSHSSSPFTRNISRDNGSRIDFILVSLKLERCIKAADILPDILGSDHCPVYS DLDILDDRIEPGTTQVPIPKFEARYKYNLRNHNVLEMFAKKDTNKESNKQKYCVSK VMNTKKNSNIKNKSLDSFFQKVNGEKDDRIKESSEIPQQAKKRISTPKLNFKDVFGK PPLCRHGEESMLKTSKTSANPGRKFWICKRSRGDSNNTESSCGFFQWVSKKKSRPN QAPSRSYNPSHSQPSATSKSSKFNKQNEERYQWLVDERDAQRRPKSDPEYDPRTLY IPSSAWNKFTPFEKQYWEIKSKMWDCIVFFKKGKFFELYEKDALLANALFDLKIAG GGRANMQLAGIPEMSFEYWAAQFIQMGYKVAKVDQRESMLAKEMREGSKGIVKR ELQCILTSGTLTDGDMLHSDLATFCLAIREEPGNFYNETQLDSSTIVQKLNTKIFGAA FIDTATGELQMLEFEDDSECTKLDTLMSQVRPMEVVMERNNLSTLANKIVKFNSAP NAIFNEVKAGEEFYDCDKTYAEIISSEYFSTEEDWPEVLKSYYDTGKKVGFSAFGGL LYYLKWLKLDKNLISMKNIKEYDFVKSQHSMVLDGITLQNLEIFSNSFDGSDKGTLF KLFNRAITPMGKRMMKKWLMHPLLRKNDIESRLDSVDSLLQDITLREQLEITFSKLP DLERMLARIHSRTIKVKDFEKVITAFETIIELQDSLKNNDLKGDVSKYISSFPEGLVEA VKSWTNAFERQKAINENIIVPQRGFDIEFDKSMDRIQELEDELMEILMTYRKQFKCS NIQYKDSGKEIYTIEIPISATKNVPSNWVQMAANKTYKRYYSDEVRALARSMAEAK EIHKTLEEDLKNRLCQKFDAHYNTIWMPTIQAISNIDCLLAITRTSEYLGAPSCRPTIV DEVDSKTNTQLNGFLKFKSLRHPCFNLGATTAKDFIPNDIELGKEQPRLGLLTGANA AGKSTILRMACIAVIMAQMGCYVPCESAVLTPIDRIMTRLGANDNIMQGKSTFFVEL AETKKILDMATNRSLLVVDELGRGGSSSDGFAIAESVLHHVATHIQSLGFFATHYGT LASSFKHHPQVRPLKMSILVDEATRNVTFLYKMLEGQSEGSFGMHVASMCGISKEII DNAQIAADNLEHTSRLVKERDLAANNLNGEVVSVPGGLQSDFVRIAYGDGLKNTK LGSGEGVLNYDWNIKRNVLKSLFSIIDDLQS*
**random-linker**-Apn2p	MSSSENT**GSASGSGD**LLDGKSENTIRFLTFNVNGIRTFFHYQPFSQMNQSLRSVFDF FRADIITFQELKTEKLSISKWGRVDGFYSFISIPQTRKGYSGVGCWIRIPEKNHPLYHA LQVVKAEEGITGYLTIKNGKHSAISYRNDVNQGIGGYDSLDPDLDEKSALELDSEGR CVMVELACGIVIISVYCPANSNSSEEGEMFRLRFLKVLLRRVRNLDKIGKKIVLMGD VNVCRDLIDSADTLEQFSIPITDPMGGTKLEAQYRDKAIQFIINPDTPHRRIFNQILAD SLLPDASKRGILIDTTRLIQTRNRLKMYTVWNMLKNLRPSNY*

PfDHFR	MMEQVCDVFDIYAICACCKVESKNEGKKNEVFNNYTFRGLGNKGVLPWKCNSLD MKYFCAVTTYVNESKYEKLKYKRCKYLNKETVDNVNDMPNSKKLQNVVVMGRT SWESIPKKFKPLSNRINVILSRTLKKEDFDEDVYIINKVEDLIVLLGKLNYYKCFIIGG SVVYQEFLEKKLIKKIYFTRINSTYECDVFFPEINENEYQIISVSDVYTSNNTTLDFIIY KKTNNKMLNEQNCIKGEEKNNDMPLKNDDKDTCHMKKLTEFYKNVDKYKINYEN

REFERENCES

1. Gibson assembly. *The Dictionary of Genomics, Transcriptomics and Proteomics* (2015), pp. 1–1.

2. O. W. Ryan, J. H. D. Cate, Multiplex engineering of industrial yeast genomes using CRISPRm. *Methods Enzymol.* **546**, 473–489 (2014).

3. O. W. Ryan, J. M. Skerker, M. J. Maurer, X. Li, J. C. Tsai, S. Poddar, M. E. Lee, W. DeLoache, J. E. Dueber, A. P. Arkin, J. H. D. Cate, Selection of chromosomal DNA libraries using a multiplex CRISPR system. *Elife*. **3** (2014), doi:10.7554/eLife.03703.

4. B. M. Hall, C.-X. Ma, P. Liang, K. K. Singh, Fluctuation analysis CalculatOR: a web tool for the determination of mutation rate using Luria-Delbruck fluctuation analysis. *Bioinformatics*. **25**, 1564–1565 (2009).

5. J. Law, F. R. Hampel, E. M. Ronchetti, P. J. Rousseeuw, W. A. Stahel, Robust Statistics-The Approach Based on Influence Functions. *The Statistician*. **35** (1986), p. 565.

6. L. Benatuil, J. M. Perez, J. Belk, C.-M. Hsieh, An improved yeast transformation method for the generation of very large human antibody libraries. *Protein Eng. Des. Sel.* **23**, 155–159 (2010).

7. R. S. McIsaac, P. A. Gibney, S. S. Chandran, K. R. Benjamin, D. Botstein, Synthetic biology tools for programming gene expression without nutritional perturbations in Saccharomyces cerevisiae. *Nucleic Acids Res.* **42**, e48 (2014).

8. J. Yuvaniyama, P. Chitnumsub, S. Kamchonwongpaisan, J. Vanichtanankul, W. Sirawaraporn, P. Taylor, M. Walkinshaw, Y. Yuthavong, Insights into antifolate resistance from malarial DHFR-TS structures. *Nat. Struct. Mol. Biol.* **10**, 357–365 (2003).

9. A. Grote, K. Hiller, M. Scheer, R. Münch, B. Nörtemann, D. C. Hempel, D. Jahn, JCat: a novel tool to adapt codon usage of a target gene to its potential expression host. *Nucleic Acids*

Res. **33**, W526-W531 (2005).

10. Afgan, Enis, et al. "The Galaxy platform for accessible, reproducible and collaborative biomedical analyses: 2018 update." *Nucleic Acids Res.* 46.W1 (2018): W537-W544.

11. Katoh, Kazutaka, et al. "MAFFT: a novel method for rapid multiple sequence alignment based on fast Fourier transform." *Nucleic Acids Res.* 30.14 (2002): 3059-3066.
