# Supplementary material for: Polymerase-guided base editing enables in vivo mutagenesis and rapid protein engineering
Source: Nat Commun. 2021 Mar 11;12:1579. doi: 10.1038/s41467-021-21876-z (PMC7952560; doi:10.1038/s41467-021-21876-z)
Supplement: Supplementary file 2 — Reporting Summary [file 41467_2021_21876_MOESM2_ESM.pdf]

nature research

Corresponding author(s): Christina Smolke

Last updated by author(s): Feb 9, 2021

Reporting Summary

Nature Research wishes to improve the reproducibility of the work that we publish. This form provides structure for consistency and transparency in reporting. For further information on Nature Research policies, see Authors & Referees and the Editorial Policy Checklist .

Statistics

For all statistical analyses, confirm that the following items are present in the figure legend, table legend, main text, or Methods section.

n/a	Confirmed	
☐	☒	The exact sample size (*n*) for each experimental group/condition, given as a discrete number and unit of measurement
☐	☒	A statement on whether measurements were taken from distinct samples or whether the same sample was measured repeatedly
☐	☒	The statistical test(s) used AND whether they are one- or two-sided<br>*Only common tests should be described solely by name; describe more complex techniques in the Methods section.*
☒	☐	A description of all covariates tested
☒	☐	A description of any assumptions or corrections, such as tests of normality and adjustment for multiple comparisons
☐	☒	A full description of the statistical parameters including central tendency (e.g. means) or other basic estimates (e.g. regression coefficient) AND variation (e.g. standard deviation) or associated estimates of uncertainty (e.g. confidence intervals)
☐	☒	For null hypothesis testing, the test statistic (e.g. *F*, *t*, *r*) with confidence intervals, effect sizes, degrees of freedom and *P* value noted<br>*Give P values as exact values whenever suitable.*
☒	☐	For Bayesian analysis, information on the choice of priors and Markov chain Monte Carlo settings
☒	☐	or hierarchical and complex designs, identification of the appropriate level for tests and full reporting of outcomes
☒	☐	Estimates of effect sizes (e.g. Cohen's *d*, Pearson's *r*), indicating how they were calculated

Our web collection on statistics for biologists contains articles on many of the points above.

Software and code

Policy information about availability of computer code

Data collection	Code used execute the MLE method for fluctuation analysis is available on GitHub at https://GitHub.com/aaroncp1an0/TRIDENT. FlowJo was used for flow cytometry data collection.
Data analysis	Code used to analyze NGS data, generate sequencing plots, and Falcor algorithm are available on GitHub at https://GitHub.com/aaroncp1an0/TRIDENT. MAFFT and Bowtie parameters are listed in supplementary information.

For manuscripts utilizing custom algorithms or software that are central to the research but not yet described in published literature, software must be made available to editors/reviewers. We strongly encourage code deposition in a community repository (e.g. GitHub). See the Nature Research guidelines for submitting code & software for further information.

Data

Policy information about availability of data

All manuscripts must include a data availability statement. This statement should provide the following information, where applicable:

- Accession codes, unique identifiers, or web links for publicly available datasets
- A list of figures that have associated raw data
- A description of any restrictions on data availability

Data availability: Data supporting the findings of this work are available within the paper and its Supplementary Information files. Raw NGS data is publicly available through NCBI under accession number PRJNA701053. Protein and primer sequences are listed in supplementary materials. The engineered yeTadA1.0 enzyme has been deposited and is available through Addgene, in anticipation of specific requests for this material (Addgene #137735, yeTadA1.0-100AA linker-T7 RNAP fusion protein).

Field-specific reporting

Please select the one below that is the best fit for your research. If you are not sure, read the appropriate sections before making your selection.

☒ Life sciences ☐ Behavioural & social sciences ☐ Ecological, evolutionary & environmental sciences

For a reference copy of the document with all sections, see nature.com/documents/nr-reporting-summary-flat.pdf

Life sciences study design

All studies must disclose on these points even when the disclosure is negative.

Sample size	No prior estimation of requisite sample size was performed. All presented data represent measurements from samples sizes of at least three biological replicates, where biological replicates (as defined in "replication" below) represent independently grown microbial cultures. Since (i) engineering was performed at the cellular level, (ii) mutation rate is a bulk measure of cellular population, and (iii) each assayed microbial culture represents a large population of individual cells, three biological replicates were sufficient for reliable measurement of changes in mutation rate at the population level.
Data exclusions	No data was excluded
Replication	All measurements of mutation rate, growth rate were repeated on independent biological samples (i.e., independent microbial cultures or strains). All attempts at replication of results presented in this study were successful. All replicates performed in this study were biological replicates, rather than technical replicates, and represent independent data points. For example, replicate microbial cultures were grown in separate containers and assayed independently from one another. Fluctuation analysis results, FACS, NGS, and viability growth rate, experiments were replicated at least three times. There was high variability in the measurements of TAA mutation rates with SHL-fusion strains, as shown in Figure 2C, and so for this experiment N was increased to N=24, the largest number allowing the experiment to still be carried out in 96-well plates. The yeast continuous evolution experiments were carried out in a highly parallel format over the course of weeks and growth results from individual wells replicated by cloning of individual sequences.
Randomization	Randomization was not relevant to this study, as the biological subjects for assays were bulk populations of microbial cells.
Blinding	Blinding was not relevant to this study, as the biological subjects for assays were bulk populations of microbial cells.

Reporting for specific materials, systems and methods

We require information from authors about some types of materials, experimental systems and methods used in many studies. Here, indicate whether each material, system or method listed is relevant to your study. If you are not sure if a list item applies to your research, read the appropriate section before selecting a response.

Materials & experimental systems

n/a	Involved in the study	
x		Antibodies
	x	Eukaryotic cell lines
x		Palaeontology
x		Animals and other organisms
x		Human research participants
x		Clinical data

Methods

n/a	Involved in the study	
x		ChIP-seq
	x	Flow cytometry
x		MRI-based neuroimaging

Eukaryotic cell lines

Policy information about cell lines

Cell line source(s)	CEN.PK2 from ATCC
Authentication	NA
Mycoplasma contamination	NA
Commonly misidentified lines (See ICLAC register)	NA

Flow Cytometry

Plots

Confirm that:

- [x] The axis labels state the marker and fluorochrome used (e.g. CD4-FITC).
- [x] The axis scales are clearly visible. Include numbers along axes only for bottom left plot of group (a 'group' is an analysis of identical markers).
- [x] All plots are contour plots with outliers or pseudocolor plots.
- [x] A numerical value for number of cells or percentage (with statistics) is provided.

Methodology

Sample preparation	Yeast cells were sorted directly from liquid culture. No antibody staining was performed.
Instrument	BD FACSaria II
Software	FlowJo
Cell population abundance	All cells were yeast, no antibody reagents were used to mark alternative cell populations.
Gating strategy	Singlet cells were gated as is standard for yeast. mCherry positive cells were gated by comparison to an mCherry negative population.

- [x] Tick this box to confirm that a figure exemplifying the gating strategy is provided in the Supplementary Information.
